# Unilateral Cleft Lip Repair With a Simple Assymetric Z-plasty

**Published:** 2018-07-02

**Authors:** Richard J. Siegel

**Affiliations:** Plastic Surgery, John A. Burns School of Medicine, University of Hawaii, Honolulu

**Keywords:** Z-plasty, unilateral cleft lip, cleft lip, congenital, principles, plastic surgery

## Abstract

**Objective:** The objective of this case report is to provide an example of the repair of a complete unilateral cleft lip using a modification of the classic Z-plasty. **Methods:** A Z-plasty for cleft lip is described that does not depend on measurements and formulas. The tissue available on the lateral lip “unit” determines the limb length of the Z: from cupid's bow to the highest point of “good” lip skin. This length is then transposed to the medial side, scribing an arc from *both* cupid's bows. Where the arcs intersect determines length and direction of the releasing incision. The angles are *not* predetermined as in a classic Z. The incisions are made “on block” through skin, muscle, and mucosa. Flap transposition uprights the isosceles triangle-shaped philtrum, aligning the cupid's bows. **Results:** All degrees of unilateral cleft lips have been successfully repaired using this technique. The operation is simple, rapid, and dependable. There is minimal bleeding, as there is no muscle dissection. On both medial and lateral sides, the muscle is transposed toward the free border, that is, downward. Achieving downward rotation of cupid's bows along with the philtrum dimple provides attractive fullness and pout to the lower part of the upper lip. Fullness and length are permanently maintained by the medially based flap under the nose. **Conclusions:** The Z-plasty is well suited for unilateral cleft lip repair. It is especially useful for wide, complete cases but is applicable to all types of unilateral cleft lips. It is a simple, fast, and stable repair.

## DESCRIPTION

A 7-month-old Filipino male patient with a complete right unilateral cleft lip underwent repair with a simple, unique modification of a Z-plasty. The result is shown 1 day postoperatively and 3 years postoperatively.

## QUESTIONS

1. In 1965, David Davies^1^ published “The Repair of the Unilateral Cleft Lip” using a “classic” Z-plasty. Why and how has the author modified the classic Z-plasty to uniquely suit and simplify the repair of the unilateral cleft lip?
2. What is the critical and fundamental principle underlying the repair of all muscular sphincters, including the orbicularis oris of the cleft lip?
3. What are the pros and cons of using a Z-plasty for cleft lip repair?
4. How does this Z-plasty compare with the Fisher^2^ repair?

## DISCUSSION

**Figure 1:**
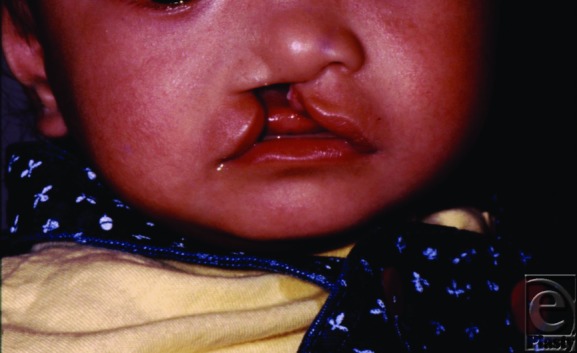
Preoperative. A 7-month-old male patient with a complete right unilateral cleft lip.

**Figure 2:**
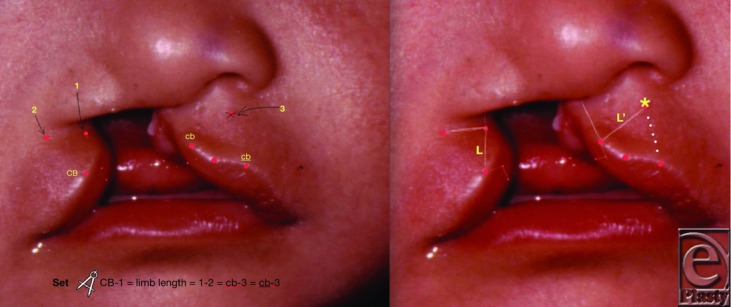
CB = cupid's bow on cleft side. 1 = highest point of good lip skin. CB-1 determines limb length of Z. 1-2 (transverse incision) is 2 mm below ala. Transpose L to medial side: CB-1 = cb-3 = cb-3. L = limb length. L’ = releasing incision. 3 and ^*^ = end of releasing incision, a point equidistant from cb and cb.

**Figure 3:**
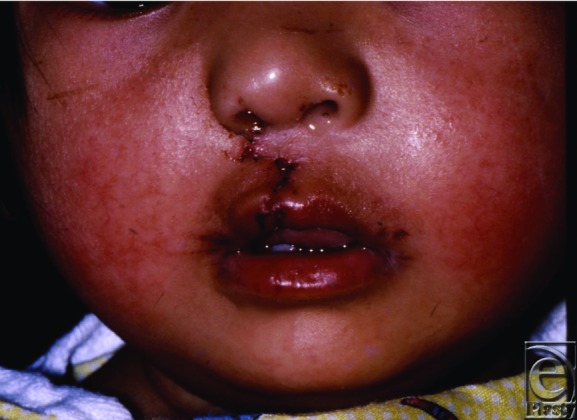
One day postoperative.

**Figure 4:**
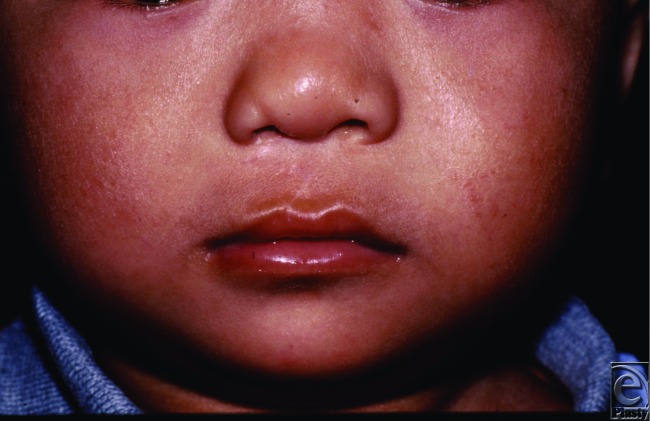
Three years postoperative.

Puzzled by the complexities of the Le Mesurier repair, Davies^1^ sought a simpler technique. His classic Z-plasty used equilateral triangles with parallel bases, the limb length chosen to provide 75% gain in length after transposition. I had observed in other Z-plasties that the actual gain in length was often slightly less than 75%. So a geometric modification was devised (Figure 2). Limb length is chosen and calipers set to the height of the lateral “unit” (CB-1). This is then scribed medially from *both* cb and cb. They cross at point 3, which determines the releasing incision (L’). The releasing incision thus ends equidistant from the 2 cupid's bows, resulting in an isosceles triangle shape. Flap transposition uprights this isosceles triangle, resulting in precisely level cupid's bows and a triangle-shaped philtrum (Figures 1,3,4).

Like the palate and eyelids, the lips form a muscular sphincter comprising concentric rings of muscle. All rings of the sphincter are *not* of equal importance. The critical rings are close to the orifice, at the mobile margin. The essential principle for dynamic sphincter repair is to move muscle from periphery to margin and lock it in place. In the palate, the Furlow double Z-plasty^3^ perfectly fulfills this principle. In the cleft lip, achieving *downward rotation of cupid's bow along with the philtrum dimple* provides attractive fullness and pout to the lower part of the upper lip. Fullness and length are permanently maintained by a flap in the periphery, that is, under the nose.

Z-plasty for cleft lip has many advantages. It is simple, with only a few key points to be marked, and easy to learn. There is no muscle dissection, as the flaps are cut “on block” through skin, muscle, and mucosa. The operation is rapidly completed, with minimal bleeding. In adults, it can be done under local anesthesia. It is ideal for revision cases, as most are short and flat, and the Z-plasty provides length and fullness. The Z avoids a straight line scar, minimizing the possibility of scar contracture and hypertrophy. Nevertheless, many cite the zig-zag scar as the main disadvantage of the Z-plasty for cleft lip repair. The complaint is that the scar crosses natural contours and does not follow the border of the anatomic subunits, that is, it is not a vertical scar.

Fisher^2^ has popularized the “anatomic subunit” or vertical scar repair. Many key points must be marked; there is a long learning curve; and wide muscle dissection is needed for closure, especially in complete clefts. On the short medial side, a small triangle allows the cupid's bow to drop, but the medial muscle (with the dimple) often must be separated from the skin, with inevitable loss or flattening of the dimple. Furthermore, the medial and lateral muscles are closed in a vertical seam, resulting in uniform tension from nasal base to vermilion border, so the lips tend to be flat, with little pout. The Z-plasty rotates medial and lateral muscles downward, toward the margin, resulting in prominent cupid's bows and lip pout. The medial (infranasal) flap locks the lip closure in position and prevents postoperative shortening. The Z-scar usually fades over time.

In summary, this “geometric” isosceles triangle Z-plasty is a unique modification of the classic Z-plasty first reported by Davies^1^ in 1965. It is a simple, fast repair, useful for all types of clefts. Like the Furlow double Z-plasty in the palate, it fulfills the paramount principle for sphincter repair: move muscle to the margin and lock it in place.
